# Gene Expression Changes as Biomarkers of Immunosenescence in Bulgarian Individuals of Active Age

**DOI:** 10.3390/biomedicines13030721

**Published:** 2025-03-15

**Authors:** Dragomira Nikolova, Yana Todorova, Zora Hammoudeh, Blaga Rukova, Radoslava Emilova, Milena Aleksova, Vesselina Koleva, Maria Nikolova

**Affiliations:** 1 Department of Medical Genetics, Medical Faculty, Medical University, 1431 Sofia, Bulgaria; blagarukova@yahoo.com; 2 National Reference Laboratory of Immunology, National Center for Infectious and Parasitic Diseases, 1504 Sofia, Bulgaria; todorova_yana@ncipd.org (Y.T.); remilova@ncipd.org (R.E.); milena.aleksova@hotmail.com (M.A.); mstoimenova@ncipd.org (M.N.); 3 Department of Clinical Laboratory, Acibadem City Clinic Tokuda Hospital, 1407 Sofia, Bulgaria; zorahammoudeh@yahoo.com (Z.H.); vessi_koleva@yahoo.com (V.K.)

**Keywords:** immunosenescence, genetic markers, chronic inflammation, adaptive and innate immune response

## Abstract

**Background/Objectives:** Immunosenescence implies innate and adaptive immunity dysfunction, which naturally occurs with aging. It is a complex multifactorial process which can be triggered by either genetic changes, immune changes or both. Numerous research studies have shown that the process of senescence goes alongside chronic immune activation. The purpose of this study is to analyze the changes in the expression of genes associated with adaptive and innate immune responses in order to identify reliable biomarkers for immune aging. **Methods**: For that aim, 55 clinically healthy individuals of active age (21–65 years) were distributed based on immunophenotyping in two groups, with and without signs of premature senescence. A gene expression analysis was subsequently made on those two groups, and the differentially expressed genes were presented and interpreted. **Results**: Altogether, forty-eight (48) genes exhibited differential expression between the two groups, most of which showed up-regulation (45) (fold change more than 2), and only three were down-regulated (fold change less than −2). The highest positive fold change showed IL-1β (10.76), BCL6 (13.25) and CCL4 (15.91), while the highest negative fold changes were documented for IL23R (−3.10), IL5 (−2.66) and PTGS2 (COX-2) (−2.15). **Conclusions**: Our results reveal that immunosenescence is positively associated with chronic inflammation, which is typical for the aging process. On the other hand, we identified markers of possible protective effects against oxidative stress and tumorigenesis. These findings can aid the early diagnosis of chronic degenerative diseases in subclinical phase, as well as the development of strategies to prevent the processes of premature immune aging.

## 1. Introduction

The steady increase in life expectancy throughout the world leads to the necessity to ensure good health and quality of life of the elderly population. In the EU, the share of the population over 65 is estimated to be 21.3%. Bulgaria has shown a constant demographic decline in the last 30 years, and by the end of 2023, the number of elderly people over 65 years was nearly 24% of the country’s population. The country has one of the highest mortality rates in Europe, partially due to the demographic aging. The cited mortality is attributable mainly to diseases of the blood circulation, neoplasms (cancer) and diseases of the respiratory system [1]. In line with these trends, the WHO has adopted a detailed Global Strategy and Action Plan on Aging and Human Health [2]. Five priority areas have been defined with an emphasis on “successful aging”, “improvement of approaches to assess, monitor and explain aging mechanisms”. In this aspect, the definition of reliable biomarkers characterizing “premature” immunological aging is a necessary basis to assess population immunological health and undertake further interventions to delay and prevent degenerative diseases associated with advanced age.

Immunosenescence is a process of innate and adaptive immunity dysfunction which naturally occurs with aging. It is a predecessor of poor vaccination efficacy, age-related diseases and neoplasms [3], susceptibility to infectious diseases and autoimmunity [4]. Chronic inflammation, referred to as “inflammaging”, and senescent T cell pool, including the reduced proportion of naïve and early memory T cells at the expense of terminally differentiated effectors with a CD28-CD57+ phenotype, are hallmarks of immunosenescence [3]. Immunosenescence is closely related to oxidative stress, described in 1961 as a condition in which the normal diploid cells “cease to proliferate after a limited number of divisions” [5]. The theory of oxidative stress explained senescence as the natural result of free radicals’ accumulation which compromises cellular functions. Later on, the idea was broadened by the concept of mitochondrial damage as the most plausible way to explain aging for the mitochondria being the organelles most vulnerable to oxidative stress [6]. Several mitochondrial miRNAs (mitomiRs) modulating organelles’ activity are involved in human inflammaging [7]. They regulate the expression of some key proteins (Bcl-2, OxPhos proteins of the electron-transport chains) thus affecting directly the production of ATP, free radicals and inflammaging.

Noteworthy, inflammaging reveals some obvious sex-related differences [8]. Women tend to live longer but experience an unhealthier lifestyle. Gender-related differences in life expectancy, disease progression and response to treatments could be explained by hormonal and genetic differences [9]. Sex differences in immune response directly affect their predisposition to age-related diseases. This results in higher vaccine efficacy and stronger innate and adaptive immune responses in women compared to men. Those differences are not only hormonal (oestrogens, progesterons and androgens), but also genetic. A very good example is TLR7, encoded by the X chromosome resulting in higher expression levels in females. On the other hand, men exhibit higher levels of natural killer cells [10]. The human X chromosome has a higher density of miRNAs when compared to autosomes; in contrast, the Y chromosome bears only 4 miRNA sequences [11].

It is important to understand that immunosenescence is a complicated process and all described possible reasons for it are interconnected. It is hypothesized that the diversity of genes might influence successful aging and longevity by modulating an individual’s response to life-threatening disorders [4]. The individual differences observed in adaptive immunity and the associated cytokine profiles logically raise the question about the extent of their genetic dependence. Genetic markers connected with senescence have been studied broadly by both international and Bulgarian research teams [12,13]. A transcriptomic study in mice revealed tissue-specific signatures of macrophage inflammaging associated with the differential expression of genes responsible for cytokines’ production, cell adhesion and antigen presentation [14].

Senescent cells can occur at any stage of an individual’s life, but definite markers of these cells are yet to be clarified. Aging is a complex process determined by multiple genetic and environmental factors, the latter of which are associated with a contemporary lifestyle, including chronic infectious and non-infectious diseases, excessive medication, unhealthy diet, environmental deterioration and stress, all leading to premature immune aging. Still, data on the altered production of some key cytokines and the role of specific genetic markers are incomplete. Moreover, there are no data on premature immune aging in healthy individuals of active age, and the genetic and epigenetic factors which determine this process. We analyzed the differential expression of immune-associated genes in people of active age in association with phenotypic signs of immunological senescence, with the aim to identify reliable biomarkers characterizing the process of premature aging. Our results will help the development of age-specific strategies for prevention and treatment, including age-adapted vaccine preparations and protocols, new antibiotics, targeted agents and anti-inflammatory foods.

## 2. Materials and Methods

### 2.1. Study Groups

This study included 55 healthy subjects (23 males and 32 females), after providing written informed consent. This study was approved by the Ethical Committee of the National Centre of Infectious and Parasitic Diseases, Sofia, Bulgaria (Institutional Review Board/Institutional Ethics Committee (IRB/IEC) number: IRB 00006384), protocol No 5/2020. All the participants were in the age range of active adults: 21–65 years (min–max), mean: 39.44 years, and reported a current professional activity. They were clinically healthy without a history of chronic diseases, hospitalization in the last 2 months, or usage of immunostimulatory or immunosuppressive drugs; they were without obesity (body mass index (BMI) < 30), with no deviations from the normal ranges for the WBC or biochemistry tests, no HIV, HSV, EBV or active CMV infection and no record of autoimmune disease, addictions or other dependencies.

### 2.2. Immunophenotyping

The share and absolute counts (AC) of lymphocytes (CD45+), T cells (CD3+), helper T cells (CD4+), cytotoxic T cells (CD8+), B-cells (CD19+) and NK cells (CD16+CD56+) were determinеd by BD Multitest 6-Color TBNK with BD Trucount Tubes (BD Bioscience, cat.# 337166). The proportion of regulatory T cells (CD4+CD25^high^CD127-, Treg), CD8+ CD57+ CD27-, CD4 and CD8 naïve (CD45RA+CCR7+), central memory (CD45RA-CCR7+), effector-memory (CD45RA-CCR7+), terminal effector (CD45RA+CCR7-) cells, as well as the expression of CD38 on CD4+ and CD8+ T cells, were determined by standard multicolor flow cytometry on a FACS Canto II flow cytometer using FACS Diva v. 6.1.3 (BD Biosciences, San Jose, CA, USA).

The following directly conjugated monoclonal antibodies were used in the multi-color flow cytometry panel: anti-hCD3 AmCyan (cat# 339186), anti-h CD4 (PE cat# 565999), anti-h CD4 APC (cat# 345771) anti-h CD25 (APC-Cy7 cat# 557753) anti-hCD45RA (FITC cat# 347723), anti-h CCR7 (PE-Cy cat# 557648), anti-h CD38 (PE cat# 345806); anti-h CD8APC (cat# 340659), anti-h CD27 (APC-H7 cat# 560222); anti-h CD127 (PcpCy5.5 cat# 560551) (BD Biosciences) anti-h CD8 (Pacific blue, cat# BL359871, Sysmex, Kobe, Japan) and anti-h CD57 (FITC cat#B49188, BeckmanCoulter, Brea, CA, USA). T lymphocyte activation was evaluated according to the number of CD38 molecules on CD4+ and CD8+ T cells. The quantitation of CD38 expression was performed using the Quantibrit PE CD38 calibration flow cytometry kit (BD Bioscience) according to the manufacturer’s instructions.

Gating strategy: lymphocytes were defined on a CD45 vs. SSC plot; the proportions of basic lymphocyte subsets (T, B, NK) were determined after plotting CD3 vs.CD19, CD3 vs. CD56+CD16, CD3 vs. CD4 and CD3 vs. CD8 expression, respectively. Naïve (CD45RA+ CCR7+), effector memory (EM, CD45RA-CCR7-), central-memory (CD45RA-CCR7+) and terminal effector (TE, CD45RA+ CCR7-) subsets were defined according to CD45RA vs. CCR7 expression within the gates of CD4+ and CD8+T cells. Treg were defined as CD25^high^CD127- cells within CD4+CD3+- gated lymphocytes. Senescent T cells were defined as CD3+CD8+- gated lymphocytes with CD57+CD27- phenotype.

### 2.3. Statistical Analysis

Between-group comparisons of quantitative values were performed by Student’s *t*-test for independent samples (for data with normal distribution), or Mann–Whitney test (for data without normal distribution). Data are presented as means (±standard deviation, SD) or medians (min, max), respectively. Categorical values (gender distribution) were compared by the chi-square test. Analyses were performed with GraphPad Prism v.9.

### 2.4. Isolation of RNA from PBMC of Each Sample, Quality Check and Preparation of cDNA

PBMC were isolated from whole blood by density-gradient media Ficoll-Paque^®^. RNA from isolated PBMC was extracted using the RNeasy Mini Kit (Cat. No./ID: 74104, Qiagen, Valencia, CA, USA) according to the manufacturer’s instructions (at sterile conditions and at 4 °C to prevent RNA degradation). Buffer RLT was supplemented with β-mercaptoethanol. Тhe RNA yield and quality were determined using a NanoDrop^®^ 8000 Spectrophotometer. After the elimination of the residual genomic DNA by DNAse I, pooled RNA samples were created using aliquot RNA amounts. The pooled samples were transcribed into corresponding cDNAs by RT-PCR using 250 ng of the purified RNA and the RT^2^ First Strand Kit (Qiagen, CA, USA).

### 2.5. Pathway-Focused Gene Expression Analysis

In the next step, cDNAs were mixed with RT^2^ SYBR Green ROX qPCR Mastermix (Cat. No. 330529, Qiagen), and the mixtures were aliquoted into the wells of an RT^2^ Profiler PCR Array Human Innate & Adaptive Immune Responses (Cat. No. PAHS-077Z, QIAGEN). RT² Profiler PCR Arrays are highly reliable and sensitive gene expression profiling tools for analyzing focused panels of genes in signal transduction, biological processes or disease research pathways using real-time PCR. RT² Profiler PCR Array system examined the expression of 84 genes simultaneously in one plaque. The arrays are pre-optimized, which ensures better results; the procedure is quick and easy to perform using a standard quantitative real-time PCR tool.

RT^2^ Profiler PCR Array Human Innate & Adaptive Immune Responses includes genes for proteins participating in innate immunity: pattern recognition receptors (*TLRs*, *NOD1*, *NOD2*, *NLRP3*, etc.), cytokines (*CCL2*, *CCL5*, *CSF2*, *CXCL10*, *IFNA1*, *IFNB1*, *IL18*, *IL1A*, *IL1B*, *IL2*, *CXCL8* and *TNF)*, other innate immunity genes (*CASP1*, *MAPK8*, *MYD88*, *NFKB1*, *STAT1,* etc.); genes of the adaptive immunity: Th1 markers (*CCR5*, *CD80*, *CXCR3*, *IL18* and *IL23A*), Th2 markers (*CCR4*, *CCR8*, *CD87*, *GATA3*, *IFNB1*, *IL10*, *IL13,* etc.), Treg markers (*FOXP3*, *IL10*, etc.), cytokines (*CCL2, CCL5, CXCL10, IFNA1*, etc.); genes for proteins participating in the inflammatory response (*APCS*, *C3*, *CCL5*, *CRP*, *IL1A* and *TNF*) and genes for defense against viruses and bacteria (*CXCL10*, *MYD88*, *IFNB1*, *IFNG* and *IFNR1*). Each array contains a panel of proprietary controls to monitor genomic DNA contamination (GDC) as well as the first strand synthesis (RTC) and real-time PCR efficiency (PPC). Reagents labeled with Sybr Green dye compatible with RT-PCR instrument are also included. The list of genes included in the RT^2^ Profiler PCR Array Human Innate & Adaptive Immune Responses, as well as their position on the PCR plate, are given in Table 1.

### 2.6. Аnalysis of the Results

After the PCR, all the built-in controls (RTC, PPC and GDC) on the array passed the validity criteria. The arithmetic means of the data from the assays for the housekeeping/reference genes listed in the table (*ACTB*, *B2M*, *GAPGH*, *HPRT1* and *RPLP0*) were used to normalize the raw data. The relative expression was determined using the data from the real-time cycler and the ΔΔCt method which is recommended in such types of analyses [15]. It is based on the determination of ΔCt of each individual gene compared to the expression of a house-keeping gene. When comparing two groups of individuals, the ΔΔCt value for each gene is calculated, which practically shows the degree of the gene expression change. The differential expression of each gene is represented by a fold change or a fold regulation. The up-regulated genes have a fold change greater than one, while the down-regulated—less than one. The analysis was performed by RT^2^ Profiler Data Analysis Software (http://www.qiagen.com/geneglobe, Qiagen, Valencia, CA, USA), and the fold regulation threshold was set to 2. Generally, fold regulation values greater than 2 have genes which we consider to be up-regulated in the test group vs. the control group, while fold regulation values less than −2 represent the genes down-regulated in the test group of individuals.

## 3. Results

### 3.1. Definition of Individuals with Senescent Immunophenotype (“Test” Group)

**Figure 1:**
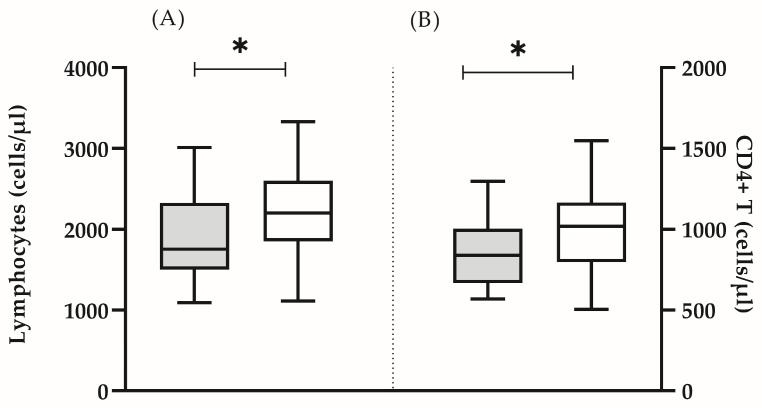
Absolute count of lymphocytes (**A**) and CD4+ T cells (**B**) in the test group (grey bars) and in the control group (white bars). The boxplot bar represents median (min–max) (* *p* < 0.05).

**Figure 2:**
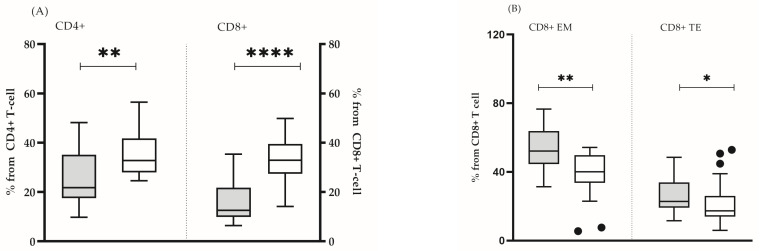
Proportions of naïve CD4+ and CD8+ T cells (**A**) and the share of effector memory and terminal effector CD8+ T cells (**B**) in test group (grey bars) and in control group (white bars). The boxplot bar represents median (min–max). (* *p* < 0.05; ** *p* ≤ 0.01; **** *p* ≤ 0.0001.)

In all the analyzed subjects, both the percentage and the absolute count of the main lymphocyte populations (T lymphocytes, B cells and NK cells) were within the reference range [16]. It was found that some clinically healthy individuals showed a significantly lower number of lymphocytes (mean ± SD 1912 ± 562 vs. 2227 ± 518, *p* = 0.05) (Figure 1A), CD4 T cells (mean ± SD 851 ± 213 vs. 1005 ± 258, *p* = 0.05) (Figure 1B), as well as naïve CD4 + (median (min–max): 21.8 (9.8–48.0) vs. 32.7 (24.6–56.0), *p* = 0.005) and CD8+ (median (min–max): 12.6 (6.4–35.3) vs. 32.9 (12.0–49.8), *p* = 0.0001) lymphocytes (Figure 2A). In this group, the lower proportion of naive CD8+ T- cells reflected a significantly higher proportion of effector memory (median (min-max): 52.1 (31.4–76.7) vs. 40.1 (5.5–54.2) *p* = 0.002) and terminally differentiated (median (min-max): 22.8 (11.6–48.5) vs. 17.4 (6.0–52.9) *p* = 0.047) CD8+ T cells (Figure 2B). In addition, we detected a higher proportion of CD57+CD8+ lymphocytes, yet, without statistical significance (median (min–max): 7.6 (1.9–48.2) vs. 8.1 (0.2–20.1), *p* = 0.206). Based on the obtained immunological results, and previously published phenotypic features of immunosenescent T cells [3], we characterized that group of individuals as “immunosenescent” (“test group”) (Table 2).

### 3.2. Gene Expression Analysis

We analyzed the expression level of 84 immune response-related genes in two pooled samples of both groups (39 individuals of the “control” group and 16 individuals of the “test” group, respectively). Among the group of individuals which are considered to be immunologically exhausted, we identified altogether 45 up-regulated genes. Of them, those with a fold regulation higher than 5 were 23. The genes with highest up-regulation values (more than 10) were IL1B (10.76), BCL6 (13.25) and CCL4 (15.91). Down-regulated genes were less in number—only three genes showed substantially decreased expression among test individuals (IL23R, IL5 and PTGS2). The list of up- and down-regulated genes is presented in Table 3.

**Figure 3:**
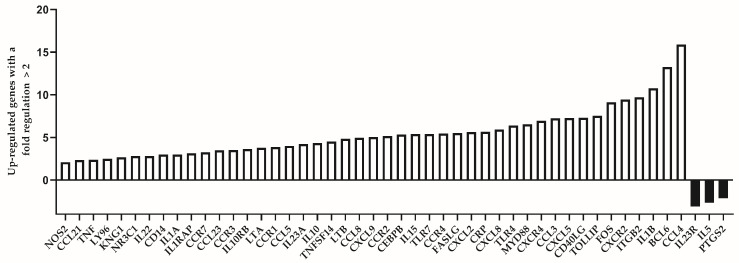
Differentially expressed genes with a fold regulation more than 2 (up-regulated) or less than −2 (down-regulated) in the group of individuals with premature immunosenescence (test group).

The differentially expressed genes between the two analyzed groups are represented visually in Figure 3. The number of the up-regulated genes is substantially higher compared to the down-regulated ones (45:3).

**Figure 4:**
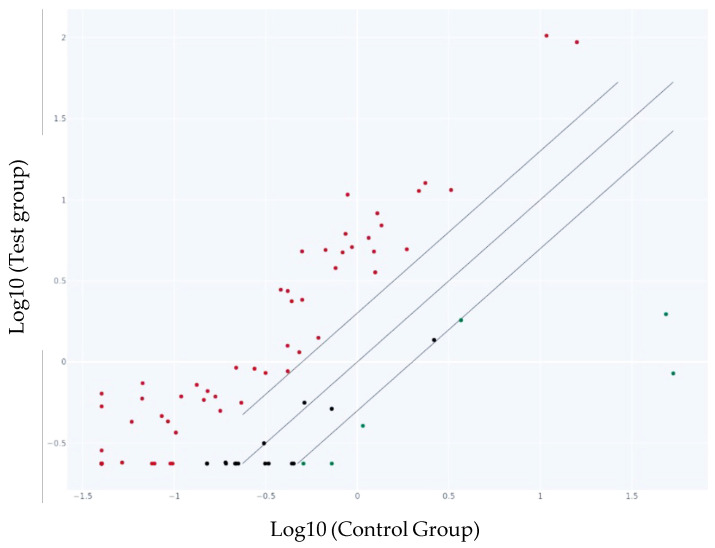
Scatter Plot of the genes in the test group vs. the control group. The center diagonal line indicates unchanged gene expression, while outer lines indicate the selected fold regulation threshold. Genes with data points beyond the outer lines in the upper left and lower right corners are up-regulated or down-regulated, respectively. The represented points are more than the presented genes in Table 2 as we selected only the genes with a fold change more than 2 and less than −2. The red dots represent the up-regulated, the black ones—genes with no change in the gene expression and the green ones—down-regulated genes.

The scatter plot graph compares the normalized expression of every gene on the PCR array between the two selected groups by plotting them against one another to visualize gene expression changes (Figure 4).

## 4. Discussion

Immunosenescence is а progressive decline in immune functions associated with increased susceptibility to age-related diseases and infections and low response to vaccines, that does not necessarily coincide with biological aging. The theories proposing different mechanisms of aging (DNA damage, mitochondrial dysfunction, thymic involution, impaired stress response etc.) finally converge to the establishment of low-grade chronic inflammation and immune activation known as “inflammaging” [17]. The neuroendocrine hypothesis defines aging as a neuronal loss of control over thypothalamic–pituitary–adrenal axis, inevitably affecting the immune cells through their hormonal receptors [18]. Triggering factors like chronic stress or recurrent viral infections (e.g., CMV infection) could accelerate aging and induce signs of premature immunosenescence in clinically healthy younger adults. The latter is characterized by a decrease in naïve at the expense of memory T cells, reduced repertoire of T- and B-cell antigen specific receptors and increased levels of proinflammatory cytokines. The immunosenescent T cells express certain phenotypic markers as CD57 combined with the loss of CD28 and CD27, and a low proliferative activity [19]. In our study, we accepted that changes at the gene expression level are an earlier and more sensitive indicator for this condition. Therefore, we studied the expression patterns of 84 immune response-related genes in healthy adults of active age showing phenotypic signs of premature immune aging.

We found substantially increased expression of three genes associated with the state of immunosenescence: *CCL4*, *BCL6* and *IL1B*. Elevated chemokines levels commonly occur with aging but the mechanism underlying this age-associated change is not fully understood. It is probably connected with the increased systemic inflammation in the body and the appearance of chronic illnesses, when chemokines direct the lymphocyte migration from the bloodstream to lymphoid organs or to sites of inflammation [20]. Previously known as macrophage inflammatory protein (MIP-1β), CCL4 is released in response to inflammation by activated leucocytes, lymphocytes, endothelial and muscle cells. Several studies on ovarian and prostate cancer show that CCL4 creates instability in the tumor microenvironment and, probably, facilitates carcinogenesis by stimulating angiogenesis and tumor progression [21]. Wang et al. showed that CCL5 (*CCL5* fold regulation 4.00 in our study) is connected with CCL4 in the process of metastasis of breast cancer cells [22]. Induced by a series of other cytokines as IL1A (*IL1A* fold regulation = 2.99), IL1B (*IL1B* fold regulation = 10.76), IL7, TNF (*TNF* fold regulation = 2.39), LPS or viral infections, CCL5 behaves like a proinflammatory cytokine. Monocytes and naïve CD8 T cells expressed higher levels of CCL4 and exhibited an age-related increase in this factor. CCL4 also could contribute to the age-related endothelial dysfunction provoked by oxidative stress and exhausted enzymatic systems of DNA repair, as well as endothelial inflammation.

Besides *CCL4*, other chemokines’ genes exhibiting higher expression levels in the prematurely aged were *CCL21*, *CCL23*, *CCL5*, *CCL8* and *CCL3* (Table 2). Recent data show their relation to chronic illnesses or injuries. Chen et al. describes CCL21 as a potential biomarker of cognitive impairment in spinal cord injury [23]. Its serum concentration is negatively correlated with the cognitive function and could be considered as an independent risk factor for cognitive impairment. Similarly to CCL21, CCL23 was associated with progression from mild cognitive impairment (MCI) to Alzheimer’s disease (AD) [24]. CCL5 (RANTES) is also a proinflammatory chemokine, mainly produced by T cells and monocytes. The CCL5/CCR5 combination has been related to specific processes in the pathogenesis of malignancies, such as angiogenesis, invasion and metastasis [25]. An interesting fact is that CCL2 (MCP-1), CCL7 (MCP-3), CCL8 and CCL13 are all known as CCR2 ligands (in our data, CCR2 fold change = 5.16). Together with CCR2, they have been detected in MS lesions [26]. A direct blocking effect of the axis CCL8-CCR8 has been demonstrated by Dangi A. et al. [27]. During allograft transplantation, donor kidney resident macrophages express a high level of CCL8, which, in turn, promotes recipient monocyte graft infiltration and the subsequent expression of CCL8. Blocking CCL8-CCR8 ensures a better short-term allograft functioning [27]. CCL3, also known as macrophage inflammatory protein 1α (MIP-1α), induces inflammatory cytokines to specific sites by binding to the CCR1/3/9 receptors [28]. Aging is related to memory deficits and CNS inflammation. CCL3 was recently shown to participate in both processes, and contribute to secondary damage after spinal cord injury [29]. It also accumulates in the bone marrow of aged mice and causes bone marrow adiposity [30].

BCL6 is an important factor in the formation of high affinity antibodies and for the functioning of B- and regulatory T cells. Germinative centers (GC) are extremely important in long-term antibody-mediated immunity. According to Fisher et al., aging was associated with accumulated Т follicular helper cells (Tfh) with a reduced expression of the transcription factor BCL6. When activated by aged antigen-presenting cells (APC), young CD4^+^ naïve T cells generate reduced numbers of activated cells with up-regulated CD40L, while Tfh cells in aging mice fail to up-regulate BCL6 expression after immunization [31]. In our data, both *BCL6* and *CD40L* were highly up-regulated in prematurely aged adults (fold change of *BCL6* = 13.25, and of *CD40L* = 7.31). This result is intriguing as it points to a key difference in the gene expression between aged individuals and individuals of immunosenescence in active age, the latter being able to stimulate the over-expression of BCL6 and CD40L. Chen Z. et al. defined BCL6 and VEGFA as “senescence regulators” [32]. Osteoarthritis (OA) progresses with age and is associated with a higher number of senescent cells in joint tissues. Cartilage cells in OA exhibit the up-regulation of both *BCL6* and *VEGFA*, and, therefore, both genes “may be used as predictive biomarkers of OA”.

Among the genes with highest expression in our data were *IL1B* (fold change = 15.91) and *IL15* (5.39), although many other genes encoding interleukins were also up-regulated (*IL10*, *IL10RB*, *IL1A* and *IL22*). IL1B levels correlate with age-related mortality in human studies. By blocking IL-1 signaling, the dysfunction of HSC could be slowed down [33]. In addition, IL1B promotes the decline of pancreatic beta cell function during the aging process [34] ultimately leading to diabetes. The plasma levels of pro-inflammatory cytokines (IL-6, TNF and IL-1β) are accepted as risk factors in cardiovascular and neurodegenerative diseases and increase in elderly with various comorbidities [35]. On the other hand, IL-15 has low levels in older individuals [36]. As IL-15 is associated with the defense against intracellular pathogens, as well as the reactivation of memory T cells, we presume that frequent and constant meeting with pathogens and the need for memory cell activation has led to its high expression in our data. It could also be associated with a mitochondrial signal for healing that generally decreases with age [37], or being overweight. In general, the up-regulated pro-inflammatory cytokine genes in our list pinpoint their key role in inflammatory aging.

The presence of significantly activated TLR genes (*TLR7*, *TLR4* and *TOLLIP*) (Table 2) in our data is in accordance with previous findings that immunosenescence affects TLRs’ function leading to higher infectious morbidity and mortality in geriatric patients [38]. TLRs are an important class of pattern-recognition receptors (PRRs), which initiate innate immune response. TLR7 (*TLR7* fold change = 5.40) induces the production of proinflammatory cytokines and interferon type I. It recognizes intracellular single-stranded RNA and promotes autoimmune diseases, such as systemic lupus erythematosus (SLE) and rheumatoid arthritis (RA), while its effects are MYD88-dependent (*MYD88* fold change = 6.56) [39]. According to Chen et al., the high expression of TLR7 and its MYD88-dependent signaling is common in adult-onset Still’s disease [40]. Excessive TLR expression may have detrimental effects: the high expression of TLR7 induces demyelination and motor impairment [41]. Additionally, a member of FAS ligand’s family FASLG (*FASLG* fold change = 5.53) has also been implicated in the progression of several autoimmune diseases (such as SLE, ALPS (autoimmune lymphoproliferative syndrome) and immunodeficiency with autoinflammation) [42]. It is supposed that the dysfunction of the FAS-FASLG pathway could be caused by gene mutations. This could be an important point of future research.

TLR4, similarly to TLR7, (*TLR4* fold change = 6.41) also activates pro-inflammatory cytokines and transcription factors NF-κB and AP-1 through MYD88. The chronic inflammatory response triggered by TLR4 contributes to the onset of age-related disorders, like Alzheimer’s disease, cancer, osteoarthritis, myocardial disorders and diabetes [43]. Toll-interacting protein TOLLIP (*TOLLIP* fold change 7.53) is an adaptor protein highly expressed in the CNS, specifically the cerebral cortex. It is associated with neurodegenerative diseases, pulmonary diseases, cardiovascular disease, inflammatory bowel disease and malignancy. Its mechanism of action is connected with autophagy and vacuole trafficking [44].

Among the overexpressed genes is also *CRP* (fold change 5.65)—a well-known marker of inflammation. Together with IL6 and TNF alpha, CRP is the most frequently studied marker of frailty [45]. Frailty, too, is a marker of aging and age-related changes in innate and adaptive immunity. Elevated CRP plasma levels are often associated with different diseases in older individuals and contribute to a higher mortality rate in the population. Highly elevated expression of CRP in our data unequivocally confirms the close link between immunological senescence and chronic systemic inflammation.

We detected only a few down-regulated genes in association with premature senescence. Senescent cells secrete inflammatory mediators that enhance tumor growth, among them being prostaglandins. PTGS2 (COX-2, cyclooxygenase 2) is a key part of the arachidonic acid cycle, involved in inflammation and pain response [46]. Several studies reported the down-regulation of *COX-2*. Ricciotti E. et al. report that the inhibition of COX-2 results in heart failure with preserved ejection fraction across zebrafish, mice and humans [47]. The down-regulation of *PTGS2* is related to abnormal immunocyte infiltration and the occurrence of interstitial lung disease in systemic sclerosis (SSc-ILD) [48]. Certain polymorphisms in *PTGS2* are involved in the pathogenesis of AD [49]. Moreover, PTGS2 acts through the NF-κB signaling pathway and vice-versa. Oxidative stress and the production of reactive oxygen species activate NF-κB and are related to a change in *COX-2* expression [50]. Therefore, the down-regulation of *COX-2* in our data could be interpreted as a protective mode against oxidative stress occurring during the aging process achieving a decrease in NF-κB activity.

IL23R is a proinflammatory protumor cytokine associated with autoimmune conditions and chronic inflammatory diseases (CID), such as inflammatory bowel disease (IBD), Crohn’s disease, axial spondyloarthritis and psoriasis (Pso). IL23 shows age-dependent rather than sex-dependent variability contributing to the individual susceptibility to IL-23 mediated inflammatory processes [51]. IL23 shows tumor-promoting effects and is highly expressed in Тreg cells of the TME (tumor microenvironment). The negative expression of *IL23R* in our data could be interpreted as a protective mechanism against tumor formation and activation of protumor Tregs.

IL5 is a cytokine produced mainly by activated Th2 cells, mast cells and eosinophils. It participates in the pathogenesis of allergic diseases and the development of eosinophilia in response to aeroallergens or parasites. IL5 could also play a role in IgA production and generation of mucosal immunity. Therefore, we suggest that the increase in the air pollution in current world, as well as the frequency of asthma or hypereosinophillic syndromes, could be associated with the decreased expression of *IL5* in our data, as an evolving evolutionary factor for protection against environmental antigens [52].

### 4.1. Potential Interventions and Therapeutic Strategies

The findings of our study imply that targeting inflammatory pathways could help mitigate immune aging. The decreased immune potential facilitates bacterial and viral virulence mechanisms and suggests new approved approaches for personalized decisions. There are developed strategies of suppression for some of the genes we have identified as up-regulated. For example, a whole class of BCL6-targeted drugs have been approved, known as BCL6 inhibitors [53]. Another way to suppress the expression is by epigenetic mechanisms, such as the usage of miRNAs. miR-144-3p inhibits cell proliferation and delays the G1/S phase transition of colorectal cancer cells [54]. Moreover, the mi-RNA 125b is a negative regulator of *CCL4* and its reduction in aging correlates inversely with the increased level of CCL4 [55]. In vitro experiments with mice show that *CCL4* knockout generates anti-aging effects, such as improved wound healing [56]. The latter finding is extremely important from a practical point of view, as a potential CCL4 targeting agent could be promising against age-related vascular and dermal effects.

Some members of the TLR family are also up-regulated in our data. In an in vitro study with mice, a treatment with TLR4 antagonist improved memory loss in aged rats, while its induction activated macrophages, fibrosis and apoptosis. This once again confirms the potential inhibition of TLR4 as a promising weapon to suppress aging and its related chronic conditions. Immune inhibitors are used to suppress low-grade inflammation. Such could be, for example, mTOR inhibitors (rapamycin). It has been shown that the administration of rapamycin and its derivatives in humans can reverse immunosenescence [57].

Some down-regulated genes could have a protective impact on the process of immunosenescence and slow it down. In such a way, the silencing of *PTGS2* (*COX-2)* by siRNA significantly decreased the frequency of post-senescence neoplastic emerging (PSNE) cells, thus showing a protective effect on keratinocytes with age. The silencing of *PTGS2* has a similar protective effect against ischemic stroke in mice, promoting the angiogenesis of endothelial progenitor cells [58]. The suppression of *IL23R* increases the responsiveness to IL12, leading to more efficient antitumor immune responses [59]. Therefore, the axis IL-23/IL-23R is meant to be a promising therapeutic target for the destabilization of Treg cells [59,60]. The decrease in IL5 controls eosinophil development, maturation and activation, and will decrease the type 2 inflammatory response [61]. The suppressed expression of IL5 will inhibit allergen-provoked airway eosinophilia and hyperreactivity.

Another possible solution is the development of novel antibiotics with higher bioactivity and cytotoxicity (like Abaucin) [62]. In addition, we may not avoid mentioning the generation of targeted therapies in the face of monoclonal antibodies for the secondary prevention of bacterial infections. A good example is Bezlotoxumab targeting toxin B of *C. difficile*. Bezlotoxumab prevents the damage of *C. difficile* on colonic mucosa by neutralizing toxin B [63].

Caloric nutritional schemes and an intake of foods with anti-inflammatory properties could also help the immune system surveillance [64]. Some macro- and micro-nutrients, as well as bioactive compounds, could positively affect the immune response. They are not only involved in molecular aspects of immunity, but also influence in a good way the gut microbiota (plan-derived fibers, prebiotics, zink, orin, vitamins, etc.) [65].

### 4.2. Limitations of the Study

A major limitation of our study is the comparison of gene expression data only, without further protein validation. In addition, this study captures just a single time frame of gene expression, while tracking of immune aging markers over time has not been performed. As immunosenescence has been associated with the emergence of chronic (respiratory or cardio-vascular) illnesses, it would be intriguing to analyze the correlation of gene expression findings with clinical outcomes in future prospective studies.

## 5. Conclusions

In the current study, we analyzed the differential expression of genes related to adaptive and innate immune responses in active people with/without signs of immunosenescence. This is the first study analyzing gene expression not in elderly but in clinically healthy individuals with premature immunosenescence. Our results undoubtfully show a higher gene expression of markers of chronic inflammation, supporting the theory of the “immune exhaustion” as a main factor of immunosenescence. In addition, the observed down-regulation of a limited number of genes could be newly interpreted as a protective reaction against oxidative stress, tumor formation and hypereosinophilia in response to an increasing number of environmental allergens. In conclusion, our study confirmed established mechanisms of aging while proposing some new interpretations of this phenomenon in individuals of active age.

## Figures and Tables

**Table 1:** List of genes included on the Qiagen plate (RT^2^ Profiler PCR Array Human Innate & Adaptive Immune Responses); PTC—positive controls; and H01 to H06—house-keeping genes.

Position	Ref Seq Number	Symbol	Description
A01	NM_001706	BCL6	B-cell CLL/lymphoma 6
A02	NM_000064	C3	Complement component 3
A03	NM_004054	C3AR1	Complement component 3a receptor 1
A04	NM_002986	CCL11	Chemokine (C-C motif) ligand 11
A05	NM_005408	CCL13	Chemokine (C-C motif) ligand 13
A06	NM_004590	CCL16	Chemokine (C-C motif) ligand 16
A07	NM_002987	CCL17	Chemokine (C-C motif) ligand 17
A08	NM_006274	CCL19	Chemokine (C-C motif) ligand 19
A09	NM_002982	CCL2	Chemokine (C-C motif) ligand 2
A10	NM_002989	CCL21	Chemokine (C-C motif) ligand 21
A11	NM_002990	CCL22	Chemokine (C-C motif) ligand 22
A12	NM_005064	CCL23	Chemokine (C-C motif) ligand 23
B01	NM_002991	CCL24	Chemokine (C-C motif) ligand 24
B02	NM_002983	CCL3	Chemokine (C-C motif) ligand 3
B03	NM_002984	CCL4	Chemokine (C-C motif) ligand 4
B04	NM_002985	CCL5	Chemokine (C-C motif) ligand 5
B05	NM_006273	CCL7	Chemokine (C-C motif) ligand 7
B06	NM_005623	CCL8	Chemokine (C-C motif) ligand 8
B07	NM_001295	CCR1	Chemokine (C-C motif) receptor 1
B08	NM_001123396	CCR2	Chemokine (C-C motif) receptor 2
B09	NM_001837	CCR3	Chemokine (C-C motif) receptor 3
B10	NM_005508	CCR4	Chemokine (C-C motif) receptor 4
B11	NM_001838	CCR7	Chemokine (C-C motif) receptor 7
B12	NM_000591	CD14	CD14 molecule
C01	NM_001250	CD40	CD40 molecule, TNF receptor superfamily member 5
C02	NM_000074	CD40LG	CD40 ligand
C03	NM_005194	CEBPB	CCAAT/enhancer binding protein (C/EBP), beta
C04	NM_000567	CRP	C-reactive protein, pentraxin-related
C05	NM_000757	CSF1	Colony-stimulating factor 1 (macrophage)
C06	NM_001511	CXCL1	Chemokine (C-X-C motif) ligand 1 (melanoma growth stimulating activity, alpha)
C07	NM_001565	CXCL10	Chemokine (C-X-C motif) ligand 10
C08	NM_002089	CXCL2	Chemokine (C-X-C motif) ligand 2
C09	NM_002090	CXCL3	Chemokine (C-X-C motif) ligand 3
C10	NM_002994	CXCL5	Chemokine (C-X-C motif) ligand 5
C11	NM_002993	CXCL6	Chemokine (C-X-C motif) ligand 6 (granulocyte chemotactic protein 2)
C12	NM_002416	CXCL9	Chemokine (C-X-C motif) ligand 9
D01	NM_000634	CXCR1	Chemokine (C-X-C motif) receptor 1
D02	NM_001557	CXCR2	Chemokine (C-X-C motif) receptor 2
D03	NM_003467	CXCR4	Chemokine (C-X-C motif) receptor 4
D04	NM_000639	FASLG	Fas ligand (TNF superfamily, member 6)
D05	NM_005252	FOS	FBJ murine osteosarcoma viral oncogene homolog
D06	NM_000619	IFNG	Interferon, gamma
D07	NM_000572	IL10	Interleukin 10
D08	NM_000628	IL10RB	Interleukin 10 receptor, beta
D09	NM_000585	IL15	Interleukin 15
D10	NM_002190	IL17A	Interleukin 17A
D11	NM_001562	IL18	Interleukin 18 (interferon-gamma-inducing factor)
D12	NM_000575	IL1A	Interleukin 1, alpha
E01	NM_000576	IL1B	Interleukin 1, beta
E02	NM_000877	IL1R1	Interleukin 1 receptor, type I
E03	NM_002182	IL1RAP	Interleukin 1 receptor accessory protein
E04	NM_000577	IL1RN	Interleukin 1 receptor antagonist
E05	NM_020525	IL22	Interleukin 22
E06	NM_016584	IL23A	Interleukin 23, alpha subunit p19
E07	NM_144701	IL23R	Interleukin 23 receptor
E08	NM_000879	IL5	Interleukin 5 (colony-stimulating factor, eosinophil)
E09	NM_000600	IL6	Interleukin 6 (interferon, beta 2)
E10	NM_000565	IL6R	Interleukin 6 receptor
E11	NM_000584	CXCL8	Interleukin 8
E12	NM_000590	IL9	Interleukin 9
F01	NM_000211	ITGB2	Integrin, beta 2 (complement component 3 receptor 3 and 4 subunit)
F02	NM_000893	KNG1	Kininogen 1
F03	NM_000595	LTA	Lymphotoxin alpha (TNF superfamily, member 1)
F04	NM_002341	LTB	Lymphotoxin beta (TNF superfamily, member 3)
F05	NM_015364	LY96	Lymphocyte antigen 96
F06	NM_002468	MYD88	Myeloid differentiation primary response gene (88)
F07	NM_003998	NFKB1	Nuclear factor of kappa light polypeptide gene enhancer in B-cells 1
F08	NM_000625	NOS2	Nitric oxide synthase 2, inducible
F09	NM_000176	NR3C1	Nuclear receptor subfamily 3, group C, member 1 (glucocorticoid receptor)
F10	NM_000963	PTGS2	Prostaglandin-endoperoxide synthase 2 (prostaglandin G/H synthase and cyclooxygenase)
F11	NM_003821	RIPK2	Receptor-interacting serine-threonine kinase 2
F12	NM_000450	SELE	Selectin E
G01	NM_001039661	TIRAP	Toll-interleukin 1 receptor (TIR) domain containing adaptor protein
G02	NM_003263	TLR1	Toll-like receptor 1
G03	NM_003264	TLR2	Toll-like receptor 2
G04	NM_003265	TLR3	Toll-like receptor 3
G05	NM_138554	TLR4	Toll-like receptor 4
G06	NM_003268	TLR5	Toll-like receptor 5
G07	NM_006068	TLR6	Toll-like receptor 6
G08	NM_016562	TLR7	Toll-like receptor 7
G09	NM_017442	TLR9	Toll-like receptor 9
G10	NM_000594	TNF	Tumor necrosis factor
G11	NM_003807	TNFSF14	Tumor necrosis factor (ligand) superfamily, member 14
G12	NM_019009	TOLLIP	Toll interacting protein
H01	NM_001101	ACTB	Actin, beta
H02	NM_004048	B2M	Beta-2-microglobulin
H03	NM_002046	GAPDH	Glyceraldehyde-3-phosphate dehydrogenase
H04	NM_000194	HPRT1	Hypoxanthine phosphoribosyltransferase 1
H05	NM_001002	RPLP0	Ribosomal protein, large, P0
H06	SA_00105	HGDC	Human Genomic DNA Contamination
H07	SA_00104	RTC	Reverse Transcription Control
H08	SA_00104	RTC	Reverse Transcription Control
H09	SA_00104	RTC	Reverse Transcription Control
H10	SA_00103	PPC	Positive PCR Control
H11	SA_00103	PPC	Positive PCR Control
H12	SA_00103	PPC	Positive PCR Control

**Table 2:** Absolute counts and proportions of the studied lymphocyte subsets.

	Healthy Individuals (“Control Group”) (*n* = 39)	Healthy Immunosenescent Individuals (“Test Group”) (*n* = 16)	*p* Value
years	37.69 ± 10.0	44.00 ± 12.65	0.09
male/female	18/21	5/11	0.47
Lymphocyte population			
Lymphocyte (AC)	2227 ± 518	1912 ± 562	0.05
CD3+CD4+ T (AC)	1005 ± 258	851 ± 213	0.05
CD3+CD8 T (AC)	492 ± 158	461 ± 188	0.44
B cells (AC)	238 (89–716)	186 (71–458)	0.17
NK cells (AC)	317 ± 163	262 ± 129	0.27
CD4/CD8	2.0 (1.1–3.7)	2.76 (1.1–3.1)	0.55
CD4+ naive	32.7 (24.6–56.0)	21.8 (9.8–48.0)	0.005
CD4+ EM	31.8 (14.1–56.3)	38.2 (5.0–55.5)	0.076
CD4+ CM	25.0 (6.1–46)	27.3 (16.3–52.0)	0.314
CD4+ TE	5.7 (1.1–19.2)	5.9 (1.4–21.8)	0.545
CD8+ naive	32.9 (12–49.8)	12.6 (6.4–35.3)	0.0001
CD8+ EM	40.1 (5.5–54.2)	52.1 (31.4–76.7)	0.002
CD8+ CM	4.0 (0.3–25.2)	4.7 (1.3–8.3)	0.360
CD8+ TE	17.4 (6.0–52.9)	22.8 (11.6–48.5)	0.047
CD8+CD57+CD27-	8.1 (0.2–21)	7.6 (1.9–48.2)	0.206
CD4+CD38ABS	3417 (1817–4481)	3662 (2568–7611)	0.241
CD8+CD38ABS	1903 (1048–2788)	2154 (1270–8351)	0.251

Statistical differences between the groups were verified with unpaired *t*-test, Mann–Whitney or Chi2 test, depending on the characteristics of the analyzed variables.

**Table 3:** Differentially expressed genes between the test group (individuals of immunological senescence) vs. the control group (individuals of no signs of immunosenescence).

Number	Up-Regulated Genes with a Fold Regulation > 2	
	Symbol	Fold Regulation
1	*NOS2*	2.10
2	*CCL21*	2.37
3	*TNF*	2.39
4	*LY96*	2.49
5	*KNG1*	2.68
6	*NR3C1*	2.81
7	*IL22*	2.83
8	*CD14*	2.99
9	*IL1A*	2.99
10	*IL1RAP*	3.14
11	*CCR7*	3.26
12	*CCL23*	3.50
13	*CCR3*	3.53
14	*IL10RB*	3.66
15	*LTA*	3.79
16	*CCR1*	3.89
17	*CCL5*	4.00
18	*IL23A*	4.24
19	*IL10*	4.34
20	*TNFSF14*	4.51
21	*LTB*	4.84
22	*CCL8*	4.97
23	*CXCL9*	5.05
24	*CCR2*	5.16
25	*CEBPB*	5.34
26	*IL15*	5.39
27	*TLR7*	5.40
28	*CCR4*	5.44
29	*FASLG*	5.53
30	*CXCL2*	5.64
31	*CRP*	5.65
32	*CXCL8*	5.92
33	*TLR4*	6.41
34	*MYD88*	6.56
35	*CXCR4*	6.94
36	*CCL3*	7.25
37	*CXCL5*	7.26
38	*CD40LG*	7.31
39	*TOLLIP*	7.53
40	*FOS*	9.11
41	*CXCR2*	9.43
42	*ITGB2*	9.70
43	*IL1B*	10.76
44	*BCL6*	13.25
45	*CCL4*	15.91
Down-Regulated Genes with a Fold Regulation < −2		
1	*IL23R*	−3.10
2	*IL5*	−2.66
3	*PTGS2*	−2.15

## Data Availability

The authors confirm that the data supporting the findings of this study are available within the article.
